# Sex Differences in Antiarrhythmic Effects of Empagliflozin: The EMPA-ICD Trial Subanalysis

**DOI:** 10.1016/j.jacasi.2025.11.017

**Published:** 2026-01-21

**Authors:** Miyo Nakano, Yusuke Kondo, Shinya Fujiki, Yuki Shiko, Yohei Kawasaki, Yoshihisa Nakagawa, Kazuyoshi Takahashi, Masaaki Okabe, Kengo Kusano, Shingen Owada, Kenichi Tsujita, Yoshiaki Kubota, Hirofumi Tomita, Toshihisa Anzai, Kenichi Iijima, Takahiro Tanaka, Yoshio Kobayashi, Tohru Minamino

**Affiliations:** a Department of Advanced Cardiorhythm Therapeutics, Chiba University Graduate School of Medicine, Chiba, Japan; b Department of Cardiovascular Medicine, Chiba University Graduate School of Medicine, Chiba, Japan; c Department of Cardiovascular Medicine, Niigata University Graduate School of Medical and Dental Sciences, Niigata, Japan; d Department of Biostatistics, Graduate School of Medicine, Saitama Medical University, Saitama, Japan; e Department of Cardiovascular Medicine, Shiga University of Medical Science, Shiga, Japan; f Department of Cardiology, Niigata City General Hospital, Niigata, Japan; g Department of Cardiology, Tachikawa General Hospital, Niigata, Japan; h Department of Cardiovascular Medicine, National Cerebral and Cardiovascular Center, Osaka, Japan; i Department of Cardiology, Iwate Medical University Hospital, Iwate, Japan; j Department of Cardiovascular Medicine, Graduate School of Medical Sciences, Kumamoto University, Kumamoto, Japan; k Department of Cardiovascular Medicine, Nippon Medical School, Tokyo, Japan; l Department of Cardiology and Nephrology, Hirosaki University Graduate School of Medicine, Aomori, Japan; m Department of Cardiovascular Medicine, Hokkaido University Graduate School of Medicine, Sapporo, Japan; n Department of Cardiovascular Biology and Medicine, Juntendo University Graduate School of Medicine, Tokyo, Japan; o Clinical and Translational Research Center, Niigata University Medical and Dental Hospital, Niigata, Japan

**Keywords:** empagliflozin, ICD, sex, type 2 diabetes, ventricular arrhythmia

## Abstract

**Background:**

The EMPA-ICD (Empagliflozin in Patients with Type 2 Diabetes Treated with an Implantable Cardioverter-Defibrillator; jRCTs031180120) trial is a prospective, multicenter, randomized, double-blind, placebo-controlled study evaluating the effects of empagliflozin on ventricular arrhythmias (VAs) in patients with type 2 diabetes (T2DM) treated with implantable cardioverter-defibrillators (ICDs). The trial showed that empagliflozin reduces VAs, but whether this effect differs by sex remains unclear.

**Objectives:**

This EMPA-ICD trial subanalysis aimed to assess sex-specific differences in the antiarrhythmic effects of empagliflozin in ICD-treated patients with T2DM.

**Methods:**

The primary endpoint was the change in the number of VAs, including nonsustained ventricular tachycardia, sustained ventricular tachycardia, and ventricular fibrillation, detected by the ICDs over 24 weeks.

**Results:**

Between April 2019 and April 2021, a total of 150 patients were randomized to receive either empagliflozin or placebo at 31 centers in Japan. In men, the rate of ventricular arrhythmia events was significantly lower in the empagliflozin group than in the placebo group, with a rate ratio of 0.33 (95% CI: 0.27-0.39; *P* < 0.001). In women, the rate ratio was 1.78 (95% CI: 0.54-5.90; *P* = 0.35), with no statistically significant difference observed. Notably, a significant interaction between sex and treatment was observed, suggesting a sex-specific treatment response (*P* = 0.006)

**Conclusions:**

The arrhythmic effect of empagliflozin in patients with T2DM treated with ICDs appears to be more pronounced in men than in women. These findings underscore the need for investigating sex-specific responses to sodium-glucose cotransporter 2 inhibitors, advancing personalized strategies for arrhythmia management.

Ventricular arrhythmias (VAs) are a major cause of syncope and sudden cardiac death (SCD), with the risk of SCD increasing in patients with reduced cardiac function owing to the underlying cardiovascular disease.^1^ Patients with type 2 diabetes (T2DM) are particularly known to face an elevated risk of both VAs and SCD.^2^, ^3^, ^4^ Large-scale clinical trials have demonstrated that sodium-glucose cotransporter 2 (SGLT2) inhibitors reduce the incidence of hospitalization for heart failure and cardiovascular death, including SCD, in patients with various conditions, including T2DM.^5^, ^6^, ^7^, ^8^, ^9^, ^10^ Notably, a meta-analysis of large randomized controlled trials demonstrated that SGLT2 inhibitors significantly reduce the risk of SCD owing to their inhibitory effect on VAs.^11^^,^^12^

Empagliflozin, a widely used SGLT2 inhibitor, has demonstrated cardiovascular benefits beyond its glucose-lowering properties, including potential antiarrhythmic effects. Specifically, the EMPA-ICD (Empagliflozin in Patients with Type 2 Diabetes Treated with an Implantable Cardioverter-Defibrillator; jRCTs031180120) trial was the first prospective, multicenter, double-blind, randomized trial designed to evaluate the antiarrhythmic effects of empagliflozin, using implantable cardioverter-defibrillator (ICD)-recorded VAs as the primary endpoint.^13^ This trial demonstrated that empagliflozin significantly reduces the number of VA events compared with placebo treatment, suggesting a potential direct or indirect antiarrhythmic benefit. However, it remains unknown whether this antiarrhythmic benefit of empagliflozin exhibits sex-based differences.

Sex-based differences are increasingly recognized as important determinants of cardiovascular outcomes and treatment response. Specifically, female patients often exhibit distinct arrhythmic substrates, including a higher prevalence of long QT syndrome, autoimmune cardiomyopathies such as cardiac sarcoidosis, and differential autonomic tone, than male patients.^14^, ^15^, ^16^ Notably, observational studies have suggested sex-specific differences in the pharmacokinetics and cardiovascular effects of SGLT2 inhibitors.^17^, ^18^, ^19^, ^20^ Understanding sex-based differences in the effect of empagliflozin is clinically important, as it may inform personalized therapeutic strategies and guide future trial design.

We hypothesized that the degree of VA suppression may be influenced by sex, reflecting underlying differences in arrhythmic substrate and drug response. Therefore, in the present prespecified subanalysis of the EMPA-ICD trial, we aimed to evaluate sex-based differences in the effect of empagliflozin on ICD-recorded VA events in patients with T2DM.

## Methods

### Study population

The present study is a subanalysis of the EMPA-ICD trial, which was a prospective, multicenter, placebo-controlled, double-blind, randomized, investigator-initiated clinical trial, involving 150 patients enrolled from 31 Japanese centers. This study included male and female patients aged 20 years or older with cardiovascular disease and T2DM who were receiving treatment with an ICD or cardiac resynchronization therapy defibrillator (CRT-D). The study was approved by the Certified Review Board of the Niigata University Graduate School of Medicine and was performed in compliance with the Declaration of Helsinki and the Clinical Trials Act. All enrolled patients provided written informed consent before eligibility screening. The sponsors were Nippon Boehringer Ingelheim Co Ltd and Eli Lilly and Company.

The Steering Committee developed the protocol and statistical analysis plan, oversaw patient recruitment, supervised data analysis, identified problems during the conduct of the study, discussed solutions, and coordinated any actions required for study operations; the Data and Safety Monitoring Board evaluated safety-related data, discussed the need to amend the protocol, considered the appropriateness of continuing the study, and made respective recommendations; and the Event Assessment Committee evaluated the data related to each arrhythmia event, considered the appropriateness of continuing the study, and made recommendations.

### Procedure

All participants who met the eligibility criteria were randomly assigned to treatment with empagliflozin or placebo at a ratio of 1:1. After randomization, patients received empagliflozin (10 mg once daily) or placebo for 24 weeks in a double-blind manner. Patients were evaluated for clinical status and adverse events at trial visits every 3 months. In patients receiving treatment for arrhythmia or underlying cardiac disease, changes in antiarrhythmic medications were minimized as much as possible during the study period. ICD/CRT-D recordings were evaluated at week 0 as the baseline and week 24 of the 24-week treatment period. Moreover, 24-hour Holter electrocardiogram monitoring and hematological and echocardiographic tests were performed before (at week 0) and after treatment (at week 24).

### Data collection

The primary endpoint was the change in the number of VAs, including nonsustained ventricular tachycardia (VT), VT, and ventricular fibrillation, recorded by the ICD/CRT-D at baseline (assessed at week 0) and the 24-week treatment period (assessed at week 24).

Secondary endpoints were as follows: 1) number of VAs recorded by ICD/CRT-D during the treatment period; 2) change in the number of antitachycardia pacing and shock therapies recorded by the ICD/CRT-D from the baseline to the treatment period; and 3) change in the number of total, single, and double ventricular premature contractions (VPCs), nonsustained VTs, and VTs, recorded by Holter monitoring before (week 0) and after treatment (week 24). Additional secondary endpoints included changes from weeks 0 to 24 in serum ketone concentrations, including acetoacetic acid, 3-hydroxybutyric acid, and total ketone bodies; and plasma catecholamine concentrations, including adrenaline, noradrenaline, and dopamine, measured in the fasting and bed-resting states. Changes from weeks 0 to 24 in glycated hemoglobin, hematocrit, brain natriuretic peptide (BNP), body weight, and systolic blood pressure were also evaluated in both groups.

### Statistical analyses

All analyses were performed according to the intention-to-treat principle, consistent with the EMPA-ICD trial.^13^ Baseline characteristics were summarized separately for men and women. Continuous variables were presented as mean ± SD and categorical variables as n (%). Group comparisons of continuous variables were performed using the Student’s *t*-test, and those of categorical variables using the chi-square test. The primary efficacy endpoint was the number of ventricular arrhythmic events recorded by the ICD or CRT-D during the 24-week treatment period. For descriptive purposes, the change in event counts from week 0 to week 24 was summarized within each group as mean ± SD. To formally compare groups while accounting for baseline event frequency, we fitted a Poisson regression model with a log link that included treatment assignment (empagliflozin vs placebo), sex, and their interaction as fixed effects. The natural logarithm of each participant’s baseline event count (week 0) was included as an offset term. The exponentiated model coefficients are presented as log rate ratios and rate ratios with 95% CIs; the significance of the treatment assignment × sex interaction term was also assessed. For secondary outcomes, change from week 0 to week 24 was calculated within each group. Between-group differences in change were compared using the Student’s *t*-test. Overdispersion in the Poisson model was assessed using Cameron and Trivedi’s dispersion test for Poisson regression. The test showed no evidence of overdispersion, and therefore the Poisson model was adopted for the primary analysis. All statistical tests were 2-sided and a *P* value <0.05 was considered statistically significant. Statistical analyses were performed using R version 4.5.0 (R Foundation for Statistical Computing).

## Results

### Baseline characteristics

Between April 2019 and April 2021, a total of 150 patients were randomized to receive either empagliflozin or placebo at 31 centers across Japan. The trial population was predominantly male (125 of 150, 83.3%). Baseline characteristics stratified by sex are summarized in Tables 1 and 2. Among male patients, 63 were assigned to the empagliflozin group and 62 to the placebo group. The empagliflozin group exhibited significantly higher glycated hemoglobin levels than the placebo group (*P* = 0.046); however, no significant differences were observed between the 2 groups in other baseline parameters (Table 1). Among female patients, 12 were assigned to the empagliflozin group and 13 to the placebo group. There were no significant differences in baseline characteristics between the 2 treatment groups (Table 2). Moreover, the baseline characteristics of male and female patients are summarized in Supplemental Table 1. Although differences were observed in smoking history and certain underlying conditions, no significant differences were noted in body mass index, BNP levels, cardiovascular history, or pharmacological treatment.

**Table 1:** Baseline Characteristics of Male Patients

	Empagliflozin (n = 63)	Placebo (n = 62)	*P* Value
Median (age), y	69.9 ± 8.4	68.8 ± 10.3	0.52
Smoking history	44 (69.8)	47 (78.3)	0.39
Indication for ICD implantation			
Primary prevention	10 (15.9)	8 (12.9)	0.83
Ventricular fibrillation	19 (30.2)	15 (24.2)	0.58
Monomorphic ventricular tachycardia	23 (36.5)	19 (30.6)	0.61
Polymorphic ventricular tachycardia	1 (1.6)	4 (6.5)	0.35
Nonsustained ventricular tachycardia	13 (20.6)	14 (22.6)	0.96
Body mass index, kg/m^2^	25.0 ± 4.1	25.6 ± 4.4	0.40
Heart rate, beats/min	70.1 ± 9.5	67.8 ± 10.3	0.21
Systolic blood pressure, mm Hg	118.8 ± 19.6	125.1 ± 20.2	0.089
Left ventricular ejection fraction, %	45.8 ± 16.7	45.0 ± 15.7	0.79
Left ventricular ejection fraction <40%	28 (45.2)	22 (37.3)	0.49
BNP, pg/mL	61 (20-156.1)	74 (28-129.4)	0.48
Glycated hemoglobin, %	7.2 ± 0.95	6.9 ± 0.50	0.046
Hematocrit, %	41.9 ± 5.2	40.9 ± 4.9	0.26
Underlying cardiac diseases			
Ischemic heart disease	31 (49.2)	32 (51.6)	0.93
Dilated cardiomyopathy	11 (17.5)	10 (16.1)	1.00
Hypertrophic cardiomyopathy	7 (11.1)	10 (16.1)	0.58
Cardiac sarcoidosis	6 (9.5)	1 (1.6)	0.13
Brugada syndrome	5 (7.9)	7 (11.3)	0.74
Long QT syndrome	0 (0)	0 (0)	
Cardiovascular history			
Atrial fibrillation	21 (33.3)	17 (27.4)	0.60
Hypertension	39 (61.9)	43 (69.4)	0.49
Dyslipidemia	47 (74.6)	41 (66.1)	0.40
Cerebrovascular disease	10 (15.9)	3 (4.8)	0.084
eGFR (mL/min/1.73 m^2^)	58.8 ± 16.5	54.2 ± 15.9	0.13
Pharmacological treatment			
Glucose-lowering therapy	37 (61.7)	34 (58.6)	0.88
Metformin	12 (20.0)	11 (19.0)	1.00
Sulfonylurea	9 (15.0)	5 (8.6)	0.43
Dipeptidyl peptidase-4 inhibitor	28 (46.7)	27 (46.6)	1.00
Glucagon-like peptide-1 agonist	1 (1.7)	0 (0)	1.00
Insulin	4 (6.7)	3 (5.2)	1.00
Other	12 (20.0)	9 (15.5)	0.69
Beta-blocker	50 (83.3)	47 (81.0)	0.93
Angiotensin-converting enzyme inhibitors or angiotensin receptor blockers	42 (70.0)	44 (75.9)	0.61
Mineralocorticoid receptor antagonists	20 (33.3)	17 (29.3)	0.79
Diuretics	26 (43.3)	26 (44.8)	1.00
Calcium-channel blocker	13 (21.7)	21 (36.2)	0.12
Antiarrhythmic drug	27 (45.0)	29 (50.0)	0.72
Cardiotonic drug	4 (6.7)	3 (5.2)	1.00
Nonpharmacological treatment			
PCI	19 (30.2)	22 (35.5)	0.66
CABG	5 (7.9)	8 (12.9)	0.54
Cardiac valve surgery	2 (3.2)	3 (4.8)	0.99
Catheter ablation	15 (23.8)	12 (19.4)	0.70
CRT-D	20 (31.7)	15 (24.2)	0.42

Variables are mean ± SD, n (%), or median (IQR).

BNP = brain natriuretic peptide; CABG = coronary artery bypass graft; CRT-D = cardiac resynchronization therapy defibrillator; eGFR = estimated glomerular filtration rate; ICD = implantable cardioverter-defibrillator; PCI = percutaneous coronary intervention.

**Table 2:** Baseline Characteristics of Female Patients

	Empagliflozin (n = 12)	Placebo (n = 13)	*P* Value
Median (age), y	67.2 ± 8.3	68.3 ± 9.9	0.76
Smoking history	3 (25.0)	3 (23.1)	1.00
Indication for ICD implantation			
Primary prevention	2 (16.7)	4 (30.8)	0.72
Ventricular fibrillation	3 (25.0)	2 (15.4)	0.92
Monomorphic ventricular tachycardia	4 (33.3)	4 (30.8)	1.00
Polymorphic ventricular tachycardia	2 (16.7)	3 (23.1)	1.00
Nonsustained ventricular tachycardia	0 (0.0)	1 (7.7)	1.00
Body mass index, kg/m^2^	25.6 ± 5.1	23.8 ± 3.6	0.34
Heart rate, beats/min	67.1 ± 8.0	67.0 ± 6.7	0.98
Systolic blood pressure, mm Hg	115.5 ± 19.2	119.3 ± 19.7	0.63
Left ventricular ejection fraction, %	53.3 ± 16.1	48.5 ± 15.7	0.47
Left ventricular ejection fraction <40%	2 (16.7)	5 (38.5)	0.44
BNP, pg/mL	100 (40–130)	88 (61–150)	0.73
Glycated hemoglobin, %	7.0 ± 0.72	7.1 ± 0.68	0.76
Hematocrit, %	38.3 ± 2.8	36.1 ± 4.2	0.14
Underlying cardiac diseases			
Ischemic heart disease	2 (16.7)	1 (7.7)	0.94
Dilated cardiomyopathy	1 (8.3)	1 (7.7)	1.00
Hypertrophic cardiomyopathy	1 (8.3)	0 (0)	0.97
Cardiac sarcoidosis	5 (41.7)	6 (46.2)	1.00
Brugada syndrome	0 (0)	0 (0)	
Long QT syndrome	2 (16.7)	1 (7.7)	0.94
Cardiovascular history			
Atrial fibrillation	3 (25.0)	3 (23.1)	1.00
Hypertension	8 (66.7)	5 (38.5)	0.31
Dyslipidemia	9 (75.0)	8 (61.5)	0.29
Cerebrovascular disease	0 (0)	1 (7.7)	1.00
eGFR (mL/min/1.73 m^2^)	51.6 ± 12.0	53.2 ± 12.4	0.75
Pharmacological treatment			
Glucose-lowering therapy	7 (58.3)	7 (53.8)	1.00
Metformin	1 (8.3)	2 (15.4)	1.00
Sulfonylurea	0 (0)	2 (15.4)	0.50
Dipeptidyl peptidase-4 inhibitor	7 (58.3)	6 (46.2)	0.84
Glucagon-like peptide-1 agonist	0 (0)	1 (7.7)	1.00
Insulin	2 (16.7)	1 (15.4)	0.94
Other	2 (16.7)	2 (15.4)	1.00
β-blocker	11 (91.7)	12 (92.3)	1.00
Angiotensin-converting enzyme inhibitors or angiotensin receptor blockers	9 (75.0)	9 (69.2)	1.00
Mineralocorticoid receptor antagonists	4 (33.3)	3 (23.1)	0.90
Diuretics	5 (41.7)	7 (53.8)	0.84
Calcium channel blocker	2 (16.7)	2 (15.4)	1.00
Antiarrhythmic drug	7 (58.3)	8 (61.5)	1.00
Cardiotonic drug	0 (0)	0 (0)	
Nonpharmacological treatment			
PCI	1 (8.3)	0 (0)	0.97
CABG	1 (8.3)	1 (7.7)	1.00
Cardiac valve surgery	1 (8.3)	2 (15.4)	1.00
Catheter ablation	3 (25.0)	1 (7.7)	0.53
CRT-D	2 (16.7)	7 (53.8)	0.14

Values are mean ± SD, n (%), or median (IQR).

Abbreviations as in Table 1.

### Primary outcome

Tables 3 and 4 summarize the mean changes in the number of VAs recorded by the ICD/CRT-D before and after treatment. Among male patients, the number of VA events was significantly reduced in the empagliflozin group from baseline to the treatment period (−2.10 events), whereas it increased in the placebo group (+2.25 events; 95% CI: −1.30 to −0.93; *P* < 0.001) (Table 3). Among female patients, the empagliflozin group experienced a slight increase in VAs (+0.55 events), whereas the placebo group demonstrated a slight decrease (−0.08 events; 95% CI: −0.62 to 1.78; *P* = 0.35) (Table 4). To formally assess treatment effects by sex, we fitted a log-linked Poisson regression model that included treatment group, sex, and their interaction, with the logarithm of the baseline count as an offset. The interaction term was significant (*P* = 0.006). Among men, the rate ratio for empagliflozin vs placebo was 0.33 (95 % CI: 0.27-0.39, *P* < 0.001), indicating a 67% reduction in arrhythmia events. Among women, the rate ratio was 1.78 (95 % CI: 0.54-5.90, *P* = 0.35), indicating no significant difference between treatments (Tables 3 and 4).

**Table 3:** Changes in the Number of Ventricular Arrhythmias Before and After Treatment in Male Patients

Empagliflozin (n = 63)	Placebo (n = 62)	Log Rate Ratio (95% CI)	*P* Value
Change from week 0 to week 24, mean (SD)	Change from week 0 to week 24, mean (SD)		
−2.10 (23.37)	2.25 (11.89)	−1.12 (−1.30 to −0.93)	<0.001

The table presents the changes in the number of ventricular arrhythmias recorded by an implantable cardioverter-defibrillator or cardiac resynchronization therapy defibrillator before treatment (baseline, assessed at week 0) and after 24 weeks of treatment (assessed at week 24) in the empagliflozin and placebo groups.

**Table 4:** Changes in the Number of Ventricular Arrhythmias Before and After Treatment in Female Patients

Empagliflozin (n = 12)	Placebo (n = 13)	Log Rate Ratio (95% CI)	*P* Value
Change from week 0 to week 24, mean (SD)	Change from week 0 to week 24, mean (SD)		
0.55 (1.81)	−0.08 (0.28)	0.58 (−0.62 to 1.78)	0.35

The table presents the changes in the number of ventricular arrhythmias recorded by an implantable cardioverter-defibrillator or cardiac resynchronization therapy defibrillator before treatment (baseline, assessed at week 0) and after 24 weeks of treatment (assessed at week 24) in the empagliflozin and placebo groups.

### Secondary outcomes

The secondary outcomes are depicted in Tables 5 and 6 for male and female patients, respectively. Among male patients, the placebo group exhibited a trend toward an increase in the number of total ventricular premature contractions, single VPCs, double VPCs, and VT episodes per day, as recorded by Holter monitoring, after treatment compared with baseline (Table 5). Notably, treatment with empagliflozin significantly increased blood acetoacetic acid levels compared with placebo (Table 5). Among female patients, empagliflozin significantly increased total blood ketones, acetoacetic acid, and 3-hydroxybutyric acid compared with placebo (Table 6). In addition, among male patients, the empagliflozin group exhibited significant decreases in glycated hemoglobin, BNP, and body weight, as well as a significant increase in hematocrit, compared with the placebo group (Table 5). However, these changes were not statistically significant for female patients (Table 6).

**Table 5:** Secondary Outcomes for Male Patients

	Empagliflozin	Placebo	*P* Value
	Change From Week 0 to Week 24, Mean (95% CI)	Change From Week 0 to Week 24, Mean (95% CI)	
Appropriate device discharge, events per 24 wk	0.07 (−0.13 to 0.27)	0.34 (−0.23 to 0.91)	0.350
Holter monitoring, events per d			
Total VPC	185.77 (−801.95 to 1,173.5)	505.15 (−383.54 to 1,393.83)	0.635
Single VPC	128.93 (−809.77 to 1067.63)	284.60 (−215.47 to 784.67)	0.781
Double VPC	−10.57 (−38.68 to 17.54)	34.17 (−79.06 to 147.4)	0.406
VT	5.42 (−7.18 to 18.02)	9.17 (−4.39 to 22.72)	0.685
Ketones, mol/L			
Total ketone bodies	100.67 (17.2 to 184.14)	10.91 (−57.78 to 79.6)	0.099
Acetoacetic acid	29.10 (8.95-49.26)	1.98 (−16.26 to 20.22)	0.048
3-Hydroxybutyric acid	71.56 (6.18-136.94)	8.94 (−42.2 to 60.07)	0.133
Catecholamines, pg/mL			
Adrenaline	0.29 (−6.3 to 6.89)	2.43 (−3 to 7.85)	0.617
Noradrenaline	−3.52 (−73.73 to 66.69)	30.60 (−39.57 to 100.76)	0.491
Dopamine	4.77 (−1.73 to 11.27)	−0.53 (−5.05 to 3.98)	0.183
Other measurements			
Glycated hemoglobin, %	−0.32 (−0.49 to −0.15)	0.07 (−0.05 to 0.18)	<0.001
Hematocrit, %	1.87 (1.1-2.63)	−0.42 (−1.37 to 0.53)	<0.001
BNP, pg/mL	−34.20 (−53.72 to −14.69)	2.79 (−13.13 to 18.71)	0.005
Body weight, kg	−2.57 (−3.21 to −1.93)	0.01 (−0.55 to 0.58)	<0.001
Systolic blood pressure, mm Hg	−2.65 (−6.22 to 0.92)	−6.26 (−10.93 to −1.59)	0.214

Variables are mean (95% CI).

VPC = ventricular premature contraction; VT = ventricular tachycardia; other abbreviation as in Table 1.

**Table 6:** Secondary Outcomes in Female Patients

	Empagliflozin Change from Week 0 to Week 24, Mean (95% CI)	Placebo Change From Week 0 to Week 24, Mean (95% CI)	*P* Value
Appropriate device discharge, events per 24 wk	0.00	0.00	—
Holter monitoring, events per d			
Total VPC	−885.22 (−2,545.4 to 774.95)	42.16 (−619.61 to 703.93)	0.198
Single VPC	−905.83 (2,544.99 to 733.33)	−385.61 (−1,412.46 to 641.25)	0.532
Double VPC	−39.49 (−116.34 to 37.35)	−14.85 (−50.44 to 20.74)	0.475
VT	−6.29 (−21.08 to 8.51)	0.15 (−0.58 to 0.89)	0.238
Ketones, μmol/L			
Total ketone bodies	270.80 (−116.67 to 658.27)	−117.85 (−277.29 to 41.59)	0.034
Acetoacetic acid	64.60 (−38.27 to 167.47)	−32.15 (−72.45 to 8.14)	0.043
3-Hydroxybutyric acid	206.20 (−80.76 to 493.16)	−85.69 (−205.16 to 33.78)	0.032
Catecholamines, pg/mL			
Adrenaline	−1.80 (−11.04 to 7.44)	1.23 (−8.75 to 11.22)	0.638
Noradrenaline	−17.60 (−168.29 to 133.09)	55.54 (−105.26 to 216.34)	0.484
Dopamine	2.70 (−7.04 to 12.44)	2.00 (−4.14 to 8.14)	0.889
Other measurements			
Glycated hemoglobin, %	−0.14 (−0.47 to 0.19)	0.00 (−0.29 to 0.29)	0.485
Hematocrit, %	1.82 (0.14-3.5)	−0.23 (−1.7 to 1.24)	0.055
BNP, pg/mL	0.51 (−48 to 49.02)	−6.18 (−52.55 to 40.19)	0.831
Body weight, kg	−1.35 (−2.91 to 0.2)	−0.03 (−1.56 to 1.5)	0.201
Systolic blood pressure, mm Hg	4.73 (−5.9 to 15.35)	−4.85 (−14.44 to 4.75)	0.155

Variables are mean (95% CI).

Abbreviations as in Tables 1 and 5.

## Discussion

Large-scale randomized controlled trials, such as DAPA-HF (Dapagliflozin and Prevention of Adverse Outcomes in Heart Failure) and EMPEROR-Reduced (Empagliflozin Outcome Trial in Patients with Chronic Heart Failure and a Reduced Ejection Fraction), have demonstrated that SGLT2 inhibitors lower the risk of hospitalization for heart failure and cardiovascular death in patients with and without diabetes.^8^^,^^9^ Meta-analyses have also suggested a potential reduction in SCD, possibly mediated through the suppression of VAs.^21^ Recent studies have also supported the antiarrhythmic effects of SGLT2 inhibitors. For instance, A subanalysis of the PROTECT (prevention of atherosclerosis by SGLT2 inhibitor: a multicentre and randomized controlled study) trial demonstrated that ipragliflozin improved left ventricular diastolic function in patients with T2DM.^22^ Improvements in diastolic function and reductions in preload may represent important mechanisms underlying the lowering of arrhythmic risk. Moreover, a prospective study using cardiac implantable electronic device monitoring confirmed that SGLT2 inhibitors were associated with a significant reduction in ventricular arrhythmic burden among patients with chronic heart failure.^23^ However, most pivotal trials have not been designed to directly assess arrhythmic outcomes, and data stratified by sex are limited. The EMPA-ICD trial was the first prospective, randomized study to evaluate the antiarrhythmic effect of empagliflozin using ICD-recorded VA events as the primary endpoint in Japanese patients with T2DM and an ICD.^13^ Although the main analysis demonstrated a significant reduction in the overall incidence of VA, the present subanalysis revealed that male patients may benefit more than female patients. The major findings of the present subanalysis of the prospective EMPA-ICD trial are that the antiarrhythmic effect of empagliflozin was more pronounced in male patients, with no similar significant reduction in VAs observed for female patients (Central Illustration). These results suggest that sex-related differences in arrhythmic substrate, comorbidities, and pharmacokinetic properties of SGLT2 inhibitors may influence treatment response.

**Central Illustration:**
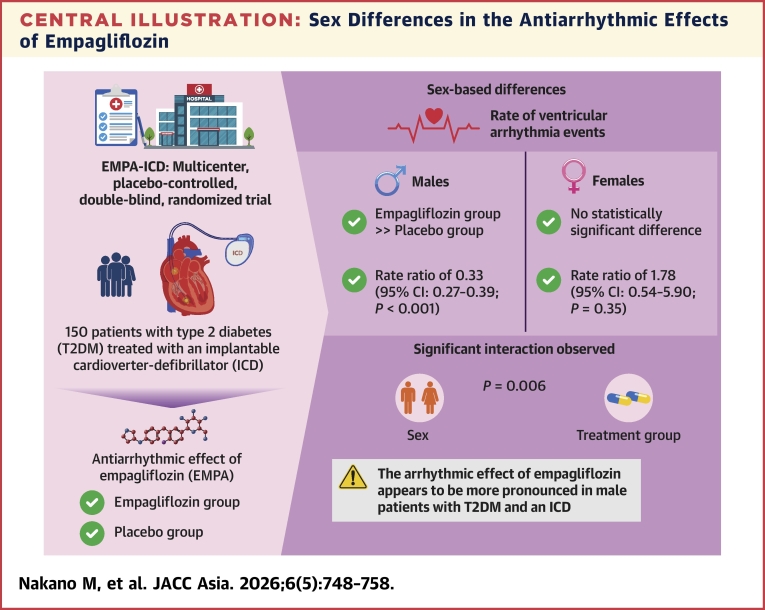
Sex Differences in the Antiarrhythmic Effects of Empagliflozin This illustration summarizes the sex-specific antiarrhythmic effects observed in the subanalysis of the EMPA-ICD (Empagliflozin in Patients with Type 2 Diabetes Treated with an Implantable Cardioverter-Defibrillator) trial of patients with type 2 diabetes mellitus (T2DM) and implantable cardioverter-defibrillators (ICDs). Empagliflozin significantly reduced ventricular arrhythmia events in male patients compared with placebo, whereas no significant reduction was observed in female patients. A significant interaction between sex and treatment (*P* = 0.006) indicates a more pronounced antiarrhythmic benefit in male patients. These findings highlight the importance of sex-based treatment response when considering sodium-glucose cotransporter 2 inhibitors for arrhythmia management.

Several mechanisms may explain the sex-specific response observed in the present study. Male patients with T2DM and cardiovascular disease generally exhibit a higher prevalence of ischemic heart disease, larger infarct scar burden, and more extensive left ventricular remodeling than female patients.^24^^,^^25^ These structural substrates may respond more favorably to the hemodynamic, metabolic, and neurohormonal benefits of SGLT2 inhibition. These benefits include reductions in preload and afterload, improvements in myocardial energetics, and attenuation of sympathetic overactivity.^26^^,^^27^ In contrast, female patients may have a higher prevalence of nonischemic cardiomyopathy, microvascular dysfunction, or inflammatory myocardial disease. In such cases, the mechanisms of VAs are less dependent on ischemic scar-related reentry and more related to repolarization instability.^28^^,^^29^ In addition, sex-specific differences in drug absorption, distribution, metabolism, and excretion have been reported for SGLT2 inhibitors.^30^ These differences may account, at least in part, for the variations in plasma exposure and efficacy. Moreover, hormonal influences, particularly those of estrogen and progesterone, may further modulate myocardial electrophysiology and arrhythmia susceptibility.^31^ Beyond the present findings, previous analyses from DAPA-HF and DELIVER (Dapagliflozin Evaluation to Improve the Lives of Patients with Preserved Ejection Fraction Heart Failure) have demonstrated broadly consistent benefits of SGLT2 inhibitors across sexes, with no significant interaction. However, some meta-analyses have suggested a numerically smaller effect size in women than in men, likely due to underrepresentation.^32^, ^33^, ^34^ In terms of arrhythmic outcomes, SGLT2 inhibitors are associated with reductions in SCD and atrial arrhythmias; however, sex-stratified data remain sparse.^35^, ^36^, ^37^ In addition, the reduction in glycated hemoglobin was significant only in male patients (Tables 5 and 6), suggesting a potential sex-related difference in the glycemic effects of empagliflozin. Although the underlying mechanism remains unclear, our findings indicate that, similar to its antiarrhythmic effect on VA suppression, empagliflozin may exert sex-specific influences on glycemic control. Further studies are warranted to elucidate the relationship between glycemic control and the present findings.

Biologically, the sex-based differences in the effect of empagliflozin may be attributed to underlying disease substrates. For example, ischemic cardiomyopathy and heart failure with reduced ejection fraction are more common in men than in women. In contrast, women frequently exhibit heart failure with preserved ejection fraction and microvascular dysfunction, conditions in which the mechanisms of VA may differ.^28^^,^^38^ Moreover, experimental data indicate sex differences in renal tubular SGLT expression and sodium handling, which may influence the natriuretic and hemodynamic responses to SGLT2 inhibition.^39^^,^^40^ Additional mechanisms include differences in autonomic modulation, metabolic substrate utilization, and effects on epicardial adipose tissue, which is reduced by SGLT2 inhibitors, potentially contributing to variations in arrhythmic risk.^41^, ^42^, ^43^

From a pharmacological perspective, population pharmacokinetic analyses do not indicate clinically meaningful differences in drug exposure between sexes.^44^^,^^45^ This suggests that the observed variation in antiarrhythmic benefit is more likely attributable to differences in clinical substrate and pathophysiology rather than drug levels. Furthermore, registry data suggest that women are less frequently prescribed SGLT2 inhibitors than men, despite similar indications, potentially contributing to exposure-related biases in outcome estimates.^46^

It also is important to consider the ethnic background of the present study population, as all participants in the EMPA-ICD trial were Japanese. Emerging evidence suggests that Asian patients with heart failure may exhibit differences in clinical phenotype, comorbidities, and arrhythmic risk compared with Western populations. For example, Asian patients develop heart failure at a younger age and have a lower body mass index, along with a higher prevalence of hypertension and diabetes, than Western patients.^47^ Furthermore, SCD accounts for a relatively higher proportion of cardiovascular mortality in Asian patients than in the Western population.^48^ Differences in electrophysiological substrate and pharmacogenomic profiles, such as drug transporter polymorphisms, may influence both arrhythmic susceptibility and drug response.^49^

In the context of SGLT2 inhibitors, registry data from East Asian countries suggest that their prescription rates remain lower than those in Europe and North America.^50^ Moreover, female patients in Asia may be particularly underrepresented in clinical trials.^51^ These disparities highlight the need for region-specific, sex-stratified analyses to confirm the reproducibility of the observed antiarrhythmic effects in broader Asian populations. Therefore, the present findings should be interpreted with caution when extrapolating to non-Japanese or multi-ethnic cohorts, highlighting the need for future multinational studies with adequate Asian representation.

The clinical implications of the present findings are 2-fold. First, they emphasize the importance of considering the sex of the patient when evaluating the expected antiarrhythmic benefit of SGLT2 inhibitors. Second, they suggest that future trials should incorporate sex-stratified analyses, as pooled results may obscure important differences in treatment response. Understanding these differences could enable more personalized approaches to arrhythmia prevention, potentially optimizing therapy and outcomes in high-risk populations. Given the pleiotropic effects of SGLT2 inhibitors, including their diuretic, anti-inflammatory, and metabolic benefits, their use may be particularly relevant for male patients with T2DM and ischemic substrates, who are at an increased baseline risk of VAs and SCD. However, the lack of a significant benefit in female patients in this study should not be interpreted as evidence of harm or lack of efficacy, but rather as a signal warranting further investigation with adequately powered, sex-stratified prospective trials.

### Study limitations

First, the number of female participants was relatively small, limiting statistical power to detect a potential treatment effect in this subgroup. Second, as the analysis was confined to Japanese patients with T2DM and an ICD, the generalizability to other ethnicities and patients without diabetes is uncertain. Third, the follow-up period of 24 weeks was relatively short. In addition, because the baseline period was defined as the 24 weeks before randomization, the exact follow-up duration for each participant could not be determined. However, considering that ICD/CRT-D data were obtained through remote monitoring for most patients, we consider that each participant was observed for almost 48 weeks, and it is unlikely that this limitation affected the study results. The long-term durability of the observed sex-specific effects remains to be established. Finally, unmeasured confounders, such as differences in the mechanism of arrhythmias, myocardial scar characteristics, and concomitant medications, may have influenced the results. In addition, previous studies have reported that women are more likely to respond to cardiac resynchronization therapy,^52^ which might have influenced the present findings. This potential effect should be acknowledged as a limitation.

## Conclusions

The present study demonstrated that the antiarrhythmic effect of empagliflozin in patients with T2DM and an ICD appears to be more evident in men than in women. This finding highlights the need for further research on sex-based differences in treatment effectiveness and pharmacokinetics and the development of treatment strategies that account for these differences.

### Availability of Data and Materials

Deidentified participant data will be made available on reasonable request 2 years after the date of publication. Requests should be directed to the corresponding author (t.minamino@juntendo.ac.jp). Requestors will be required to sign a data access agreement to ensure the appropriate use of the study data.

## Funding Support and Author Disclosures

This study was funded by Nippon Boehringer Ingelheim Co Ltd and Eli Lilly and Company and by AMED-CREST (JP20gm1110012) and Moonshot Research and Development Program (21zf0127003s0201). The funders of the study had no role in the design and conduct of the study; the collection, management, analysis, and interpretation of the data; the preparation, review, or approval of the manuscript; and the decision to submit the manuscript for publication. Dr Nakano belongs to an endowed department funded by Abbott Medical Japan Co, Ltd, Biotronik Japan Co, Ltd, and Fukuda Denshi Co, Ltd Dr Kondo has received lecture fees from Daiichi-Sankyo Co, Ltd, Bayer Pharmaceutical Co, Ltd, Abbott Medical Japan Co, Ltd, Biotronik Japan Co, Ltd, Boston Scientific Japan Co, Ltd, Japan Lifeline Co, Ltd, and Medtronic Japan Co, Ltd, as well as research funds from Daiichi-Sankyo Co, Ltd and Boston Scientific Japan Co, Ltd. Dr Fujiki received funding support for the present manuscript from Nippon Boehringer Ingelheim Co, Ltd and Eli Lilly and Company. Dr Nakagawa received grants and speaking honoraria from Nippon Boehringer Ingelheim Co, Ltd Dr Kusano received lecture honoraria from Medtronic Japan Co, Ltd and Nippon Boehringer Ingelheim Co, Ltd. Dr Tsujita received scholarship funding from Nippon Boehringer Ingelheim Co, Ltd. Dr Tomita received grants from Medtronic Japan Co, Ltd, Fukuda Denshi Kita-tohoku Hanbai Co, Ltd, Biotronik Japan Co, Ltd, Japan Lifeline Co, Ltd, and Boston Scientific Japan Co, Ltd, as well as speaking honoraria from Nippon Boehringer Ingelheim Co, Ltd Dr Anzai received honoraria from Daiichi-Sankyo Co, Ltd, Nippon Boehringer Ingelheim Co, Ltd, Bayer Pharmaceutical Co, Ltd, AstraZeneca Co, Ltd, and Novartis Pharma Co, Ltd; clinical research grants from Abbott Medical Japan Co, Ltd, Otsuka Pharmaceutical Co, Ltd, Japan Lifeline Co, Ltd, Boston Scientific Japan Co, Ltd, Nippon Boehringer Ingelheim Co, Ltd, and Daiichi-Sankyo Co, Ltd; and scholarship funds from Biotronik Japan Co, Ltd, Medtronic Japan Co, Ltd, Win International Co, Ltd, Medical System Network Co, Ltd, Hokuyaku Takeyama Holdings, Inc., and Terumo Co, Ltd. Dr Kobayashi received lecture fees from Abbott Medical Japan Co, Ltd, Bayer Japan, Bristol-Myers Squibb, Nippon Boehringer Ingelheim Co, Ltd, and Daiichi-Sankyo Co, Ltd, as well as scholarship funds from Takeda Pharmaceutical, Abbott Medical Japan Co, Ltd, Terumo, Otsuka Pharmaceutical, Nippon Boehringer Ingelheim Co, Ltd, Astellas Pharma Inc., Daiichi-Sankyo Co, Ltd, Win International Co, Ltd, Japan Lifeline Co, Ltd, and Nipro Co, Ltd Dr Minamino received funding support for the present study, study drug provision, and lecture remuneration from Nippon Boehringer Ingelheim Co, Ltd and Eli Lilly and Company. All other authors have reported that they have no relationships relevant to the contents of this paper to disclose.
