# Supplementary material for: Sex Differences in Antiarrhythmic Effects of Empagliflozin: The EMPA-ICD Trial Subanalysis
Source: JACC Asia. 2026 Jan 21;6(5):748–58. doi: 10.1016/j.jacasi.2025.11.017 (PMC13153910; doi:10.1016/j.jacasi.2025.11.017)
Supplement: Supplemental Table [file mmc1.docx]

**Supplemental Table 1.** **Baseline characteristics of male and female patients.**

| Characteristic | Male  (N=125) | Female  (N=25) | *P* value |
| --- | --- | --- | --- |
| Median age, mean (SD) | 69.3 ± 9.4 | 67.8 ± 9.0 | 0.45 |
| Smoking history, n (%) | 91 (74.0) | 6 (24.0) | <0.001 |
| Indication for ICD implantation |  |  |  |
| Primary prevention | 18 (14.4) | 6 (24.0) | 0.37 |
| Ventricular fibrillation | 34 (27.2) | 5 (20.0) | 0.62 |
| Monomorphic ventricular tachycardia | 42 (33.6) | 8 (32.0) | 1.00 |
| Polymorphic ventricular tachycardia | 5 (4.0) | 5 (20.0) | 0.013 |
| Non-sustained ventricular | 27 (21.6) | 1 (4.0) | 0.075 |
| Body mass index | 25.3 ± 4.3 | 24.7 ± 4.4 | 0.50 |
| Heart rate (bpm) | 68.9 ± 10.0 | 67.0 ± 7.2 | 0.37 |
| Systolic blood pressure (mmHg) | 121.9 ± 20.0 | 117.5 ± 19.1 | 0.31 |
| Left ventricular ejection fraction (%) | 45.4 ± 16.1 | 50.8 ± 15.8 | 0.13 |
| Left ventricular ejection fraction <40% | 50 (41.3) | 7 (28.0) | 0.31 |
| BNP (pg/ml) | 124.8 ± 149.5 | 134.7 ± 133.6 | 0.78 |
| Glycated hemoglobin (%) | 7.09 ± 0.78 | 7.07 ± 0.68 | 0.90 |
| Hematocrit (%) | 41.4 ± 5.1 | 37.1 ± 3.7 | <0.001 |
| Underlying cardiac diseases |  |  |  |
| Ischemic heart disease | 63 (50.4) | 3 (12.0) | 0.001 |
| Dilated cardiomyopathy | 21 (16.8) | 2 (8.0) | 0.42 |
| Hypertrophic cardiomyopathy | 17 (13.6) | 1 (4.0) | 0.31 |
| Cardiac sarcoidosis | 7 (5.6) | 11 (44.0) | <0.001 |
| Brugada syndrome | 12 (9.6) | 0 (0) | 0.23 |
| Long QT syndrome | 0 (0) | 3 (12.0) | 0.002 |
| Cardiovascular history |  |  |  |
| Atrial fibrillation | 38 (30.4) | 6 (24.0) | 0.69 |
| Hypertension | 82 (65.6) | 13 (52.0) | 0.29 |
| Dyslipidemia | 88 (70.4) | 17 (68.0) | 1.00 |
| Cerebrovascular disease | 13 (10.4) | 1 (4.0) | 0.53 |
| eGFR (ml/min/1.73m^2^) | 56.6 ± 16.3 | 52.5 ± 12.0 | 0.24 |
| Pharmacological treatment |  |  |  |
| Glucose-lowering therapy | 71 (60.2) | 14 (56.0) | 0.87 |
| Metformin | 23 (19.5) | 3 (12.0) | 0.55 |
| Sulfonylurea | 14 (11.9) | 2 (8.0) | 0.84 |
| Dipeptidyl peptidase-4 inhibitor | 55 (46.6) | 13 (52.0) | 0.79 |
| Glucagon-like peptide-1agonist | 1 (0.8) | 1 (4.0) | 0.78 |
| Insulin | 7 (5.9) | 3 (12.0) | 0.52 |
| Other | 21 (17.8) | 4 (16.0) | 1.00 |
| β-blocker | 97 (82.2) | 23 (92.0) | 0.36 |
| Angiotensin-converting enzyme inhibitors or angiotensin receptor blockers | 86 (72.9) | 18 (72.0) | 1.00 |
| Mineralocorticoid receptor antagonists | 37 (31.4) | 7 (28.0) | 0.93 |
| Diuretics | 52 (44.1) | 12 (48.0) | 0.89 |
| Calcium channel blocker | 34 (28.8) | 4 (16.0) | 0.29 |
| Antiarrhythmic drug | 56 (47.5) | 15 (60.0) | 0.36 |
| Cardiotonic drug | 7 (5.9) | 0 (0) | 0.46 |
| Non-pharmacological treatment |  |  |  |
| PCI | 41 (32.8) | 1 (4.0) | 0.007 |
| CABG | 13 (10.4) | 2 (8.0) | 1.00 |
| Cardiac valve surgery | 5 (4.0) | 3 (12.0) | 0.26 |
| Catheter ablation | 27 (21.6) | 4 (16.0) | 0.72 |
| CRT-D | 35 (28.0) | 9 (36.0) | 0.70 |

Variables are mean ± SD or n (%).

Body mass index is calculated as the weight (kg) divided by the square of the height (m).

BNP = brain natriuretic peptide; CABG = coronary artery bypass graft; CRT-D = cardiac resynchronization therapy defibrillator; eGFR = estimated glomerular filtration rate; ICD = implantable cardioverter-defibrillator; PCI = percutaneous coronary intervention.
